# Effect of Temperature on Oil–Water Separations Using Membranes in Horizontal Separators

**DOI:** 10.3390/membranes12020232

**Published:** 2022-02-17

**Authors:** Tao Zhang, Chenguang Li, Shuyu Sun

**Affiliations:** 1 Computational Transport Phenomena Laboratory, Division of Physical Science and Engineering, King Abdullah University of Science and Technology, Thuwal 23955-6900, Saudi Arabia; tao.zhang.1@kaust.edu.sa; 2 China National Oil and Gas Exploration and Development Company Limited, Beijing 100034, China; lichenguang@cnpcint.com

**Keywords:** oil–water separation, horizontal separator, membrane, phase-field model

## Abstract

The effect of temperature on oil–water separations is studied in this paper, focusing on the changed penetration velocities of water droplets on the separation membrane in a horizontal separator. A compact numerical scheme is developed based on the phase-field model, and the temperature effect is first theoretically analyzed regarding the key thermodynamic properties that may affect the separation performance. The computational scenario is designed based on practical horizontal separators in the oil field, and the droplet motions in the oil–water two-phase flow are simulated using our scheme under various operation conditions. It was found that a higher temperature may result in a faster penetration of the water droplets, and a larger density difference in the oil–water system is also preferred to accelerate the separation using membranes. Furthermore, increasing the operation temperature is proved to benefit the separation of water and heavy oil.

## 1. Introduction

Oil–water two-phase flow is commonly seen throughout the entire process of crude oil production and transportation [1]. The long history of oil recovery and the application of various secondary and tertiary recovery techniques have brought a higher water cut to the production in many oil fields [2], and such a problem is a general challenge faced by certain middle and late stages of reservoir development [3]. The existence of water in oil production is not preferred in the petroleum industry due to the higher risk of equipment corrosion caused by electro-chemical reactions, which are often believed to be a consequence of water–steel interactions [4]. The severe corrosion phenomenon and subsequent problems including pipe fractures and breakages may cause a leakage of oil through transportation and during storage, which may further result in a number of environmental pollution and social safety problems [5]. Furthermore, the water in oil production is also a valuable resource for certain oil fields lacking water resources, for example, in the typical desert area in the Middle East and on offshore platforms [6]. Thus, water is expected to be separated after oil gathering in the field, and then collected for further purification and injection. Moreover, oil–water two-phase flow is also a problem in other industries including machinery manufacturing and food processing [7], while oil is considered a form of pollution and should be separated before pure water usage and emission. Oil-containing wastewater may cause great harm to the environment [8]. For example, oil floating on the sea’s surface and rapidly spreading to form an impermeable oil film will hinder the re-oxygenation of the water body, thus affecting the growth of marine plankton and destroying the marine ecological balance [9]. Ultimately, the development of oil–water separation technology is closely related to the sustainable development of many economic and social fields and the long-term protection of the ecological environment. Improvements to separation efficiency and the discovery of the separation mechanisms are a key and difficult problem that we urgently need to resolve.

The current popular oil–water separation techniques mainly include the gravity method, the centrifugal method, the air flotation method, the adsorption method, the chemical method, the biological method, and the membrane separation method. Among them, membrane separation has the advantages of low energy consumption, high single-stage separation efficiency, a flexible and simple process, low environmental pollution, and strong versatility [10]. However, the application efficiency of membrane separation is affected by internal factors such as the membrane’s pollution resistance, thermal stability, and chemical stability. Operation conditions, including liquid passing velocity and injection direction, have also been detected to show an impact on the separation performance. The long history of development and extensive attraction arising from industrial demands have brought us a number of different separation membrane designs, which were proposed for various working scenarios with different water/oil droplet size distributions and operation conditions, including ultrafiltration membranes, microfiltration membranes, nanofiltration membranes, and many others. A new design of an oil–water separation membrane was proposed in [11], focusing on the special wettability that can be divided into super-hydrophobic and super-lipophilic membranes and super-hydrophilic and super-oleophobic membranes. When the oil–water mixture touches the surface of the superhydrophobic and super-lipophilic membrane, the oil droplets quickly spread and penetrate the membrane surface. The water cannot wet the membrane surface, and it is trapped on the membrane and cannot penetrate it. This idea was named the “oil-removing” method, and has been followed by a series of studies expanding its application and improving its performance [12,13,14]. However, due to the potential lipophilicity of general membrane materials, oil droplets and other impurities can easily be irreversibly adsorbed on the membrane surface in large quantities. The membrane may thus be seriously polluted, and the passing flux decays quickly. During the cleaning process, emulsified oil droplets can coalesce easily and spread on the membrane surface, causing the membrane to be poorly reusable and affected by secondary pollution. A directly opposite way is to prepare a super-hydrophilic and super-oleophobic membrane, as the so-called water-removing method. The membrane is super-oleophobic in air or water and exhibits extremely low adhesion to oil, with a rolling contact angle of only 2°~3°, effectively preventing the adhesion of oil droplets. When the oil–water two-phase fluid contacts the membrane surface, water can continuously penetrate downward, and the surface always maintains super oleophobicity. The oil is trapped on the surface, and due to the potential oil repellency of the membrane, the oil can never contaminate the membrane surface. Thus, such a design is a true anti-pollution, low-energy-consumption, long-life, and high-efficiency separation membrane, thus becoming the main development direction of oil–water separation membranes in the future [15,16].

The current studies on oil–water separation membranes focus on laboratory experiments to optimize the membrane material and examine the separation performance under various working conditions. The effects of cross-flow velocity, temperature, pressure, and surfactant concentration on permeation flux were investigated in [17], and it was pointed out that nonionic surfactant solutions have a higher permeation flux than anionic surfactant solutions. It was further discovered in [18] that, due to the adsorption of surfactants, the concentration polarization layer of the film was thickened, which may significantly reduce the permeation flux. An ultrafiltration model containing diffusion/convection effects in the membrane pores was developed in [19], which successfully fitted the permeation flux of the experimental membrane. In order to slow down the attenuation of permeation flux caused by membrane fouling, various approaches have been proposed, including improving the hydrophilicity of the membrane [20], constructing a hydrophilic polymer structure on the membrane surface [21], generating a self-cleaning surface [22], and many others. A number of numerical studies have also been reported in recent years focusing on multi-phase flow simulations informing the separations in the field [23,24], and a breakthrough model of the interfaces was proposed in [25] to enhance the understanding of phase separation mechanisms. However, practical oil–water separation in the petroleum industry requires a comprehensive understanding of both the membrane separation mechanism and the optimal operations in the field to achieve a better separation performance. In particular, the effect of various working conditions on the multi-phase flow in large oil–water separators is essentially relevant with the permeation flux, pressure, and other factors that have been proved to be effective in the membrane separation mechanism. Thus, an oil–water two-phase flow model will be developed in this paper, with a quick solver to meet engineering demands, to simulate the droplet motions in the oil–water separators, and to investigate the effect of temperature and other factors on permeation status into the membrane surface.

**Figure 1:**
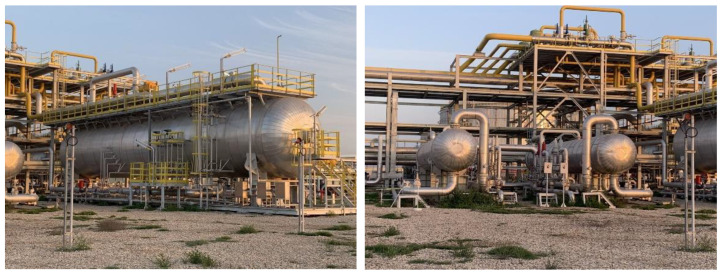
The side view and front view of the practical oil–water separator studied in this paper.

**Figure 2:**
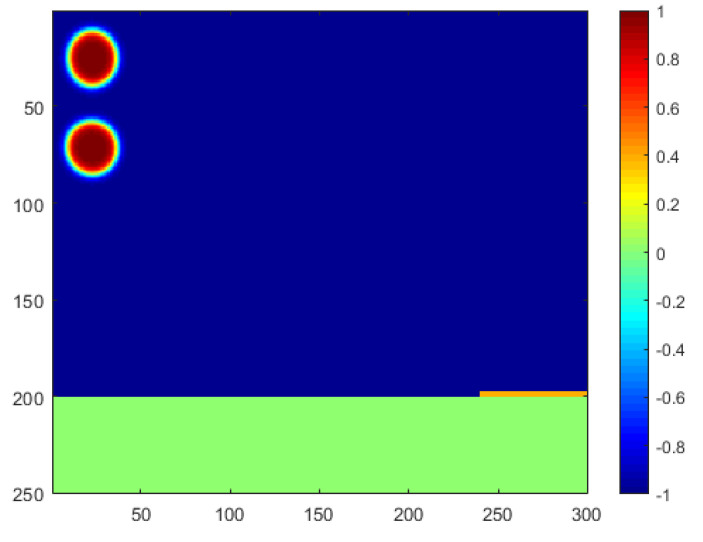
Schematic diagram of the computational domain.

The computational scenario used in this paper is generated based on practical horizontal oil–water separators commonly seen in the oil field. Photos of the side view and front view of the example separator are shown in Figure 1. As shown in the photos, the oil–water two-phase fluid gathered from production will be injected from the upper-left inlet of this separator, and a collection pipeline will be connected from the lower-right outlet to collect the separated water for further utilization. For instance, the water can be further purified and injected back into the reservoir to enhance oil recovery, which is highly preferred by our industrial partners in the desert areas of Iraq [26]. Considering the difference in density, the oil pipeline is connected with the upper-right outlet to collect the purified oil for further storage and transportation. In addition to the three main pipelines, a number of small pipelines are also attached to the separator to transport hot or cold water inside the separator wall so as to adjust the operation temperature of the separations. The corresponding computational domain in this paper is then designed as shown in Figure 2. Two oil droplets are injected from the upper-left corner, and the horizontal domain with a mesh size of 200×300 is filled with oil. The phase index is defined as 1 for the water phase and −1 for the oil phase. As a phase-field model, the phase interface can be captured with a certain thickness represented by a phase index between −1 and 1. A super-hydrophilic and super-oleophobic membrane is placed at the lower-right corner of the domain, represented by yellow, which will allow only the water phase to penetrate. The membrane has extremely low adhesion to oil, which can ensure a direct large-area contact between the water droplets and membranes so as to improve the separation efficiency. Additionally, a collection pipeline is placed under the domain, represented by green, to collect the separated water and transport it for further usage.

This paper was designed to generate a numerical scheme to simulate an oil–water two-phase fluid flow based on the phase-field model and then apply it to the investigation of practical oil–water phase separation processes. The effect of temperature on the droplet motions penetrating the filtering membrane is analyzed both theoretically and numerically. Different density ratios between oil and water are also investigated regarding the separation process. The remainder of this paper is organized as follows. A compact numerical scheme is developed in Section 2 based on the phase-field model to calculate oil–water two-phase flow problems with a fast solver. A theoretical analysis of the temperature effect on droplet motions is also carried out. The simulation results re illustrated in Section 3 with an analysis of the effect of temperature and other factors to suggest the optimal operations for oil industries. Concluding remarks are given in Section 4 to point out potential further developments continuing in this direction.

## 2. A Compact Numerical Scheme Based on the Phase-Field Model

In this section, we will first present the detailed two phase flow simulation scheme based on the phase-field model with a fast solver to meet the engineering demand. The mass conservation and energy decay property of the developed phase-field model are mathematically proved to ensure divergence and consistency of our simulation. A thermodynamic analysis of the effect of temperature on the interfacial tension and chemical potential is also carried out to theoretically suggest the optimal operation temperature of oil–water separations. 

### 2.1. Phase-Field Model

A phase-field variable ϕ is defined as an index representing the phase distributions in the domain as a function of time t and space x, which can be written as [27]:

(1) $$\phi \left(x,t\right)=\{\begin{array}{cc} 1 & \mathrm{fluid}\;1,\\ -1 & \mathrm{fluid}\;2. \end{array}$$

In this paper, fluid 1 is the water phase, and fluid 2 is the oil phase. ϕ can be either 1 or −1 for the bulk phase of the system, but also a value between −1 and 1 if located within the interface, defined as a thin (but nonzero-thickness) transient layer. We define the total free energy F(ϕ) of this two-phase system as the summation of the bulk phase free energy $F_{b}\left(\phi \right)$ and the gradient contribution $F_{\nabla }\left(\phi \right)$, which can be calculated as:

(2) $$F_{b}\left(\phi \right)=\int_{\Omega }f_{b}\left(x\right)dx,$$

(3) $$F_{\nabla }\left(\phi \right)=\int_{\Omega }\frac{c_{I}}{2}{\left|\nabla \phi \right|}^{2}dx,$$

A simple way to formulate the bulk phase free energy density $f_{b}\left(x\right)$ is to construct a polynomial function with two minimums within the two bulk phase regions, as:

(4) $$f_{b}\left(\phi \right)=\frac{c_{b}}{4}{\left(\phi^{2}-1\right)}^{2}.$$

Thus, the total free energy can be written as:

(5) $$F=F_{b}+F_{\nabla }=\int_{\Omega }\left(\frac{c_{b}}{4}{\left(\phi^{2}-1\right)}^{2}+\frac{c_{I}}{2}{\left|\nabla \phi \right|}^{2}\right)dx.$$

In order to meet the second law of thermodynamics (decaying of the total free energy), the following conservation equation is defined:

(6) $$\frac{\partial \phi }{\partial t}=-\nabla \cdot J.$$

The parameter J can be calculated using the following equation to ensure J=0 at equilibrium with a constant chemical potential:

(7) J(x)=−M∇μ,

M is the mobility coefficient and the chemical potential μ can be calculated by $\mu =\frac{\delta F}{\delta \phi }=c_{b}\left(\phi^{3}-\phi \right)-c_{I}\nabla^{2}\phi$.

The developed phase-field model can be proved to be mass-conservative by no diffusive flux on the domain boundary in the phase separation process, as:

(8) $$\frac{d\left(\int_{\Omega }\phi \left(x,t\right)dx\right)}{dt}=\int_{\Omega }\frac{\partial \phi }{\partial t}dx=\int_{\partial \Omega }J\cdot nds=0,$$

Additionally, the unconditional stability property can also be proved by the energy decay, as [28]:

(9) $$\frac{\partial F}{\partial t}=\langle \frac{\partial \phi }{\partial t},\frac{\delta F}{\delta \phi }\rangle =\langle \nabla \cdot \left(M\nabla \frac{\delta F}{\delta \phi }\right),\frac{\delta F}{\delta \phi }\rangle \;=-\langle M\nabla \frac{\delta F}{\delta \phi },\nabla \frac{\delta F}{\delta \phi }\rangle =-M{\left\|\nabla \frac{\delta F}{\delta \phi }\right\|}^{2}=-M{\left\|\nabla \mu \right\|}^{2}\leq 0.$$

### 2.2. A Compact Coupled Scheme

The stress tensor can be split into an isotropic term and an anisotropic term, as:

(10) $$\sigma =\sigma_{\mathrm{iso}}+\sigma_{\mathrm{aniso}},$$

where the two contributions can be calculated as:

(11) $$\sigma_{\mathrm{iso}}=\left(c_{I}\left(\nabla \phi \cdot \nabla \phi \right)-p_{x}\right)I,$$

(12) $$\sigma_{\mathrm{aniso}}=-c_{I}\nabla \phi ⊗\nabla \phi .$$

Recall Cauchy’s equation of motion, $\rho \frac{Dv_{i}}{Dt}=\rho g_{i}+\frac{\partial \sigma_{ij}}{\partial x_{j}}$ can help derive the following single-phase Navier–Stokes momentum equation:

(13) $$\rho \left(\frac{\partial v}{\partial t}+v\cdot \nabla v\right)=\rho g-\nabla p+\eta \nabla^{2}v.$$

The anisotropic term of stress tensor $\sigma_{\mathrm{aniso}}$, resulting from the gradient contribution of free energy and controlling the effect of interfacial tension on the two-phase flow, can be incorporated into Equation (13), to formulate the two-phase Navier–Stokes–Cahn–Hilliard coupled equation as:

(14) $$\rho \left(\frac{\partial v}{\partial t}+v\cdot \nabla v\right)=\rho g-\nabla p+\eta \nabla^{2}v-\nabla \cdot \left(c_{I}\nabla \phi ⊗\nabla \phi \right).$$

It is easy to see that $\nabla \cdot \left(\nabla \phi ⊗\nabla \phi \right)=\nabla \phi \nabla^{2}\phi +\nabla \left(\frac{\nabla \phi \cdot \nabla \phi }{2}\right)$ and the term $\left(C_{I}\nabla \phi \cdot \nabla \phi /2\right)$ can be absorbed into the pressure so that the effect of the interfacial tension on the two-phase flow can be modeled as:

(15) $$\nabla \cdot \sigma_{\mathrm{aniso}}=-c_{I}\nabla \phi \nabla^{2}\phi .$$

Recall that $\mu =\mu_{b}-c_{I}\nabla^{2}\phi$ and $\mu_{b}\nabla \phi =\nabla f_{b}$, implying $\mu \nabla \phi =\nabla f_{b}-c_{I}\nabla \phi \nabla^{2}\phi$, and we can further simplify the two-phase flow model as:

(16) $$\rho \left(\frac{\partial v}{\partial t}+\left(v\cdot \nabla \right)v\right)=\rho g-\nabla p+\eta \nabla^{2}v+\mu \nabla \phi ,\;\;\mathrm{in}\;\Omega .$$

The transport of the interface is modeled by the Cahn–Hilliard equation (with convection) as:

(17) $$\frac{\partial \phi }{\partial t}+v\cdot \nabla \phi =M\nabla^{2}\mu ,\;\;\mathrm{in}\;\Omega .$$

Coupling the conservation equation,

(18) ∇·v=0,  in Ω,

the comprehensive two-phase flow model is generated. A fast algorithm for solving the phase-field model is proposed in [27], constituted by an analytical solution as an initial guess and an accelerated matrix solver, the Shift-Matrix method. This algorithm is also applied in this paper to meet engineering demands for fast calculations for large-scale practical scenarios.

### 2.3. Effect of Temperature

#### 2.3.1. Effect of Temperature on Interfacial Tension

We first recall that the mechanical work needed to increase a surface is δW=FdL=σdA, with A being the surface area. From the following Gibbs equation,

(19) $$dU=\delta Q+\delta W=TdS-pdV+\overset{M}{\underset{i=1}{\sum }}\mu_{i}dN_{i}+\sigma dA,$$

the interfacial tension can be easily derived to be $\sigma ={\left(\frac{\partial U}{\partial A}\right)}_{S,V,N}.$ Similarly, the other following representations of interfacial tension can be derived from the other forms of Gibbs equations, as:

(20) $$\sigma ={\left(\frac{\partial H}{\partial A}\right)}_{S,p,N},\;\;\sigma ={\left(\frac{\partial F}{\partial A}\right)}_{T,V,N},\;\;\mathrm{or}\;\;\sigma ={\left(\frac{\partial G}{\partial A}\right)}_{T,p,N}.$$

Based on the previous definition, the following algebraic equation to calculate interfacial tension can be easily obtained:

(21) $$\sigma =\frac{1}{A}\left(G-\overset{M}{\underset{i=1}{\sum }}N_{i}\mu_{i}\right)=\frac{1}{A}\left(U-TS+pV-\overset{M}{\underset{i=1}{\sum }}N_{i}\mu_{i}\right),$$

which indicates a direct relationship between temperature and interfacial tension. In fact, there have been a number of empirical formulations calculating the interfacial tension as a function of temperature. For example, the following Eötvös rule [29] was proposed to describe the correlation between the interfacial tension and temperature:

(22) $$\sigma =kv^{-\frac{2}{3}}\left(T_{c}-T\right),$$

where $T_{c}$ is the critical temperature, v is the molar volume, and k is a constant often chosen to be $2.1\times 10^{-7}\;J/\left(K\cdot mol^{2/3}\right)$. Looking at the units alone, this may suggest that k is proportional to $k_{B}N_{A}^{2/3}$, where $k_{B}$ denotes the Boltzmann constant and $N_{A}$ denotes the Avogadro constant. Another commonly used formula for the interfacial tension calculation is the Guggenheim–Katayama equation [30], which can be written as:

(23) $$\sigma =\sigma^{o}{\left(1-\frac{T}{T_{c}}\right)}^{n},$$

where $\sigma^{o}$ is a variable for different species.

All of the above thermodynamic theoretical analyses and experimental empirical formulas have denoted the negative correlation between temperature and interfacial tension. As a result, a higher temperature is preferred in the oil–water phase separation process, as the lower interfacial tension will consequently help the demulsification mechanism. However, in our scheme, the chemical potential is selected to control the anisotropic stress term (in Equation (16)) instead of the interfacial tension; thus, we will derive the correlation between temperature and chemical potential in the next subsection.

#### 2.3.2. Effect of Temperature on Chemical Potential

Similar to the Gibbs equation, chemical potential can be written as partial derivatives of free energies, as:

(24) $$\mu_{i}={\left(\frac{\partial U}{\partial N_{i}}\right)}_{S,V,N_{\neq i}}={\left(\frac{\partial H}{\partial N_{i}}\right)}_{S,p,N_{\neq i}},$$

or

(25) $$\mu_{i}={\left(\frac{\partial F}{\partial N_{i}}\right)}_{T,V,N_{\neq i}}={\left(\frac{\partial G}{\partial N_{i}}\right)}_{T,p,N_{\neq i}}.$$

Thus, the following equation can easily obtain that

(26) $${\left(\frac{\partial \mu_{i}}{\partial T}\right)}_{P,n_{k}}={\left[\frac{\partial }{\partial T}{\left(\frac{\partial G}{\partial n_{i}}\right)}_{T,P,n_{j}\neq n_{i}}\right]}_{P,n_{k}}={\left[\frac{\partial }{\partial n_{i}}{\left(\frac{\partial G}{\partial T}\right)}_{P,n_{k}}\right]}_{T,P,n_{j}\neq n_{i}}\;=-{\left(\frac{\partial S}{\partial n_{i}}\right)}_{T,P,n_{j}\neq n_{i}}=-S_{i,m}.$$

As clearly indicated in the above equation, under a certain pressure and compositions, the change in chemical potential in one system is equivalent to the negative of the system per molar entropy. According to the second law of thermodynamics, the system entropy cannot be negative, which may result in a decrease in chemical potential if the temperature increases. Thus, the effect of temperature on the oil–water separation process is simulated by changing the chemical potentials in our numerical scheme accordingly in the following simulations.

## 3. Simulation Results

The Navier–Stokes–Cahn–Hilliard coupled scheme is numerically solved in this section, using the compact Shift-Matrix method to enable a fast engineering computation in a large domain defined as 250×300 meshes. The Shift-Matrix method, proposed in [27], is applied in a finite difference scheme to benefit the discretization and convergence of the numerical solver, and the numerical stability has been tested in detail in [25]. The simulation is implemented by coding in Matlab, which is a high-level integrated language by which the Shift-Matrix scheme can be easily established using the built-in functions. The horizontal separator takes the space of 200×300 meshes, filled with oil fluid and illustrated by the blue color as shown in Figure 2. The green domain, representing the water-collection pipeline, takes the space of 50×300 meshes and is located under the separator domain. The super-hydrophilic and super-oleophobic membranes are placed at the lower-right corner of the separator domain, with a size of 1×50 meshes representing a flow boundary only for the water phase. 

**Figure 3:**
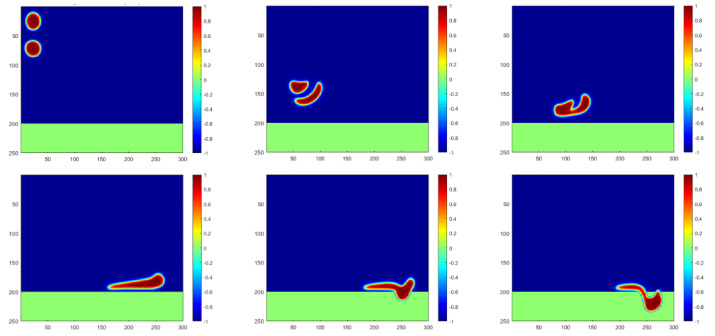
Simulation of water droplet motions at time steps of 0, 450, 700, 1500, 1650 and 1750, respectively.

The motions of the water droplets, injected from the upper-left inlet, are simulated and plotted in Figure 3 for a general case. Under the effect of gravity, water droplets with a density higher than that of the oil fluid fall down in the separator as they simultaneously flow to the right together with the oil flow. A clear reshaping can be captured on the oil–water interface, and the two droplets can merge together after certain reforming and motions. The merged big droplet may continue to flow to the right and start penetrating through the membrane towards the water injection pipeline when contacting the membrane meshes. It should also be noted that the phase-field model is adapted in this paper; thus, the interface with a certain thickness will always take some space. To benefit the plotting and understanding, the separation mechanism is defined to be effective when the interface area contacts the membrane meshes.

In the following two subsections, the effect of temperature and oil density are investigated numerically using our scheme. Five different cases are defined as shown in Table 1. Droplet motions and penetration velocity in Case 1, Case 2 and Case 3 will be compared to show the temperature effect, in which the oil density is kept as 0.8, proportional to the water density. The temperature in Case 2 is the highest, followed by that in Case 1, and the temperature in Case 3 is lowest. The effect of oil density is observed in Section 3.2 by comparing the droplet motions and penetration velocities in Case 1, Case 4, and Case 5. The operating temperature remains the same in all three cases, but the oil density is largest in Case 5, followed by Case 1, and the oil density in Case 4 is the smallest. In other words, a larger oil–water density difference is observed in Case 4, and a smaller difference is observed in Case 5. 

### 3.1. Effect of Temperature

Temperature is one of the most commonly seen operation conditions that industrial partners are concerned about. Particularly for the oil fields located in the Middle East, the environmental temperature can be greater than 323.15K in the hottest summers and remain larger than 303.15K in the coolest winters. Such extremely hot weather may result in a special operation scenario with a large environmental temperature, and the separation efficiency is often questioned under these circumstances, as well as other daily production and maintenance operations.

**Figure 4:**
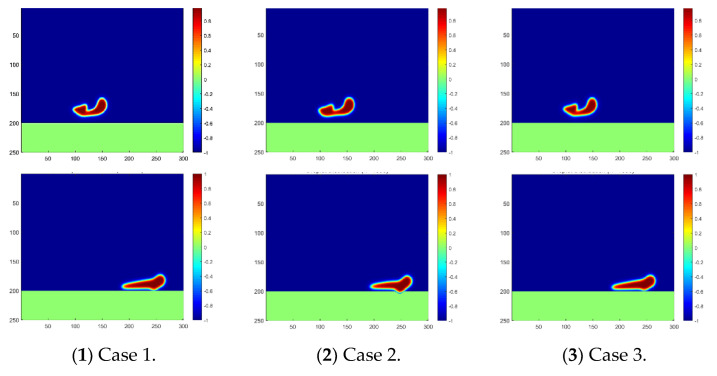
Droplet fusion and penetration into the membrane under different temperatures. The phase distribution at the time step of 800 is plotted in the first line, and at the time step of 1800, it is plotted in the second line.

As analyzed in Section 2, the temperature effect is reflected by the change in chemical potential in our numerical scheme, and a negative correlation was proved. Based on this, the droplet motions in Cases 1, 2 and 3 are simulated using our proposed scheme with various chemical potentials, and the results are plotted in Figure 4. Droplet coalescence can be captured near the time step of 800 in all three cases, with a slight difference in the shape of the fused big droplet. However, a clear difference can be observed in the contact and penetration of the water droplets into the membrane near the time step of 1600.

**Figure 5:**
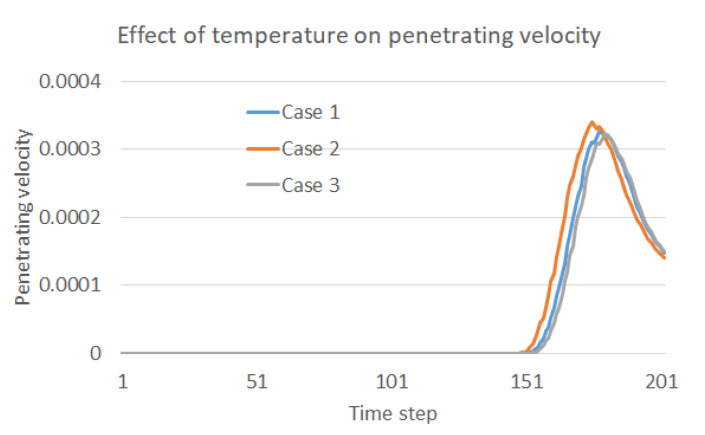
Effect of temperature on the penetration velocity into the membrane.

The contact area of the water droplets on the membrane varies during the same time step under various operation temperatures as shown in Figure 4. It can be reasonably further inferred that the penetrating moment and velocity should also vary in the three cases. To validate this inference, the penetration velocity during the whole process is recorded and compared as shown in Figure 5. As clearly indicated in the figure, the water droplet in Case 2 contacts the membrane first, and the penetration velocity is the highest. Additionally, the water droplet in Case 3 contacts the membrane at the latest moment, and the penetration velocity is the lowest. Thus, it has been proved that a higher temperature is preferred to benefit the oil–water separation in the sense of accelerating the contact and penetration of the water droplets into the separation membrane. Moreover, the penetration velocity increases first and then decreases after reaching the peak, which reflects the decay in falling speed during the second stage of droplet penetration.

### 3.2. Effect of Oil Density

Oil density is another key property of production in the oil field, which may change with recovery time and techniques. For example, heavier oil can be successfully produced if the more advanced secondary and tertiary oil recovery techniques are applied. Generally speaking, oil production in a certain block is always easier at the beginning with lighter oil, but heavy oil cannot be wasted in later development.

**Figure 6:**
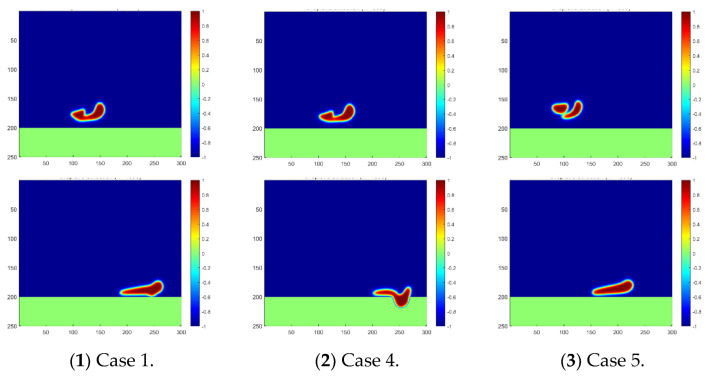
Droplet fusion and penetration into the membrane for different oil densities. The phase distribution at the time step of 800 is plotted in the first line, and at the time step of 1600, it is plotted in the second line.

**Figure 7:**
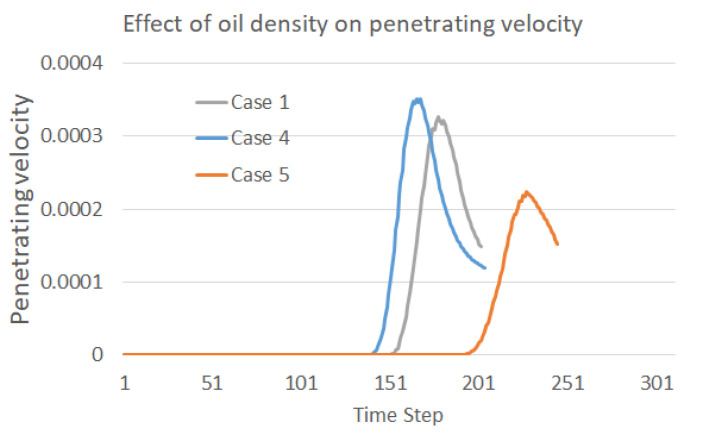
Effect of temperature on the penetration velocity into the membrane.

The effect of oil density is easily detected, as shown in the Figure 6, in which the falling, coalescence, and penetration of the droplet are significantly slowed down during our simulation if the oil density is increased. The two water droplets may not even merge together at the time step of 800 in Case 5 with the largest oil density, and may also not contact the separation membrane at the time step of 1600. These phenomena explain the difficulties often met in oil fields when purifying the produced heavy oil with water. In particular, the recovery of heavy oil always involves the injection of water in various conditions, for example, the popular SAGD approach (Steam-Assisted Gravity Drainage), which results in a non-ignorable content of water in the production. The obstacles to oil–water separation caused by a high oil density can also be observed from the penetration velocity curve plotted in Figure 7. It can be easily inferred from the plot that the droplets move slowly under the circumstance of a small difference between the oil and water densities. This finding makes it reasonable to conclude that gravity is the main driving force behind droplet motion in this scenario.

**Figure 8:**
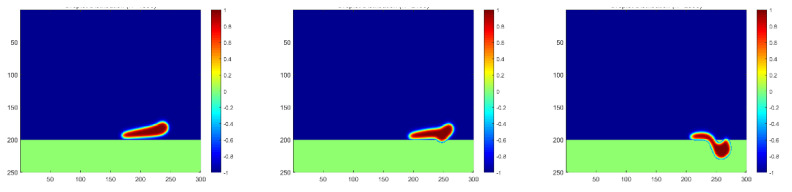
Droplet penetration into the membrane for a high oil density at the time steps of 1800, 2100, and 2300, respectively.

As indicated in the *x*-axis in Figure 7, the droplets’ contact with the membrane can only be captured if the observation is extended to more time steps. The penetration process is plotted in Figure 8, showing a much smaller moving and penetration velocity, which meets exactly the lower peak of the curve of Case 5. Moreover, a slower increase in the first stage and a slower decrease in the second stage both indicate a longer separation process of water droplets passing the membrane, which should be considered in the study of membrane mechanisms.

**Figure 9:**
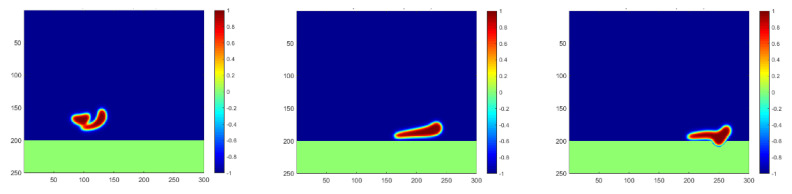
Droplet coalescence and penetration into the membrane for a high oil density under a higher temperature at the time steps of 800, 1700 and 2100, respectively.

The simulation and analysis above point out the improvement to the separation efficiency the operation temperature is increased. Thus, a direct resolution to enable an efficient oil–water separation for heavy oil is to increase the temperature in the horizontal separator. In order to validate the effectiveness of this intuitive idea, a new case, Case 6, is defined in Table 2, with the oil density set as 0.9 and the temperature set as T2. The droplets’ coalescence and penetration in this case are plotted in Figure 9, with a clear acceleration as compared with that in Figure 8. The two droplets may merge earlier if the temperature is increased, and a faster penetration through the membrane can be accomplished. 

**Figure 10:**
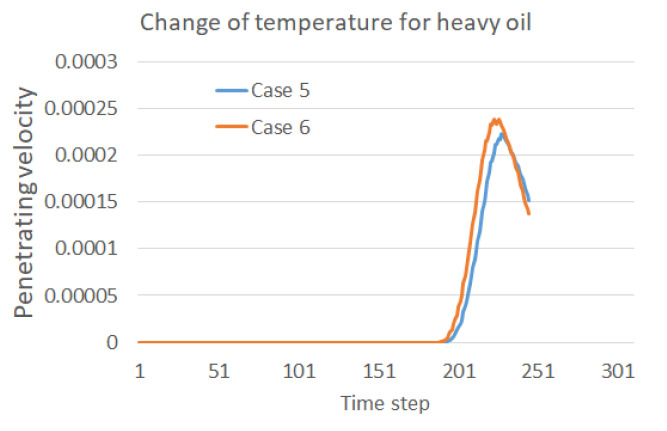
Effect of temperature on the penetration velocity into the membrane for the heavy oil.

The improved oil–water separation for heavy oil with a higher operation temperature can also be illustrated by comparisons with the penetration velocities, as shown in Figure 10. The water droplets reach the membrane area faster in Case 6, and then rapidly increase to a higher peak. A faster decrease is also observed after the peak compared with the lower temperature case, which also validates the faster passing of the whole merged water droplet. Comparisons between Figure 5, Figure 7 and Figure 10 may suggest that the engineers operating oil–water separations in the field can consider the effect of temperature and density ratio to control the time of water droplet motions in the separator in order to improve the separation efficiency and manage the collection of separated water.

In practice, engineers can increase the operation pressure in the separator if the detected oil density is increased so as to keep a continuous penetration time and velocity. Thus, the effectiveness of our numerical approach is validated by the positive feedback from our industrial partner using our approach to optimize the separation and collection of the separated water.

## 4. Discussion and Conclusions

Oil–water separation is an important process in the energy industry by which to ensure safe and efficient oil production. The membrane separation method stands out among a number of separation technologies, and super-hydrophilic and super-oleophobic membranes are a current research hotspot after their development of more than 20 years. Previous studies have mainly focused on the separation mechanisms and the effect of various environmental conditions on the laboratory experimental separation tests. However, practical separation in the oil field is often challenged by various operation temperatures and oil densities at different locations in different recovery stages. The energy industry is more interested in the falling and coalescence processes of water droplets in large-scale separators, which may reflect further the penetration of the droplets into the membrane and the corresponding separation efficiency. Thus, a computational scenario is designed in this paper based on the real horizontal separators in the oil field. A compact numerical scheme is developed with good mass conservation and energy decay properties. The effect of temperature on separation is analyzed first, taking into account the changing chemical potential and interfacial tension. Droplet motions in the horizontal space are simulated using our algorithm, and the effect of temperature and oil density is investigated thoroughly. It has been found that an increase in operation temperature may accelerate the droplet motions and penetration through the membrane, while a high oil density is not preferred for efficient oil–water separations. A good solution is then proposed, in which we introducing an improvement to separation performance by increasing the temperature, which is then validated to benefit oil–water separation for heavy oil.

**Figure 11:**
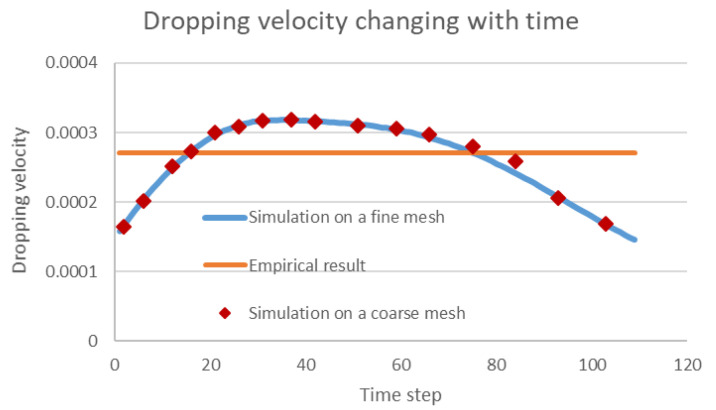
Comparison between the simulated dropping velocities and empirical results.

One main advantage of the numerical simulation for solving multiphase flow problems is its general adaptability in complex engineering scenarios where laboratory experiments are hard to implement. As the common oil–water separator in the field is not transparent, the droplet motions in the separator are not visible for validation of the numerical result. However, our numerical scheme can be validated by checking the simulated dropping velocity of one single droplet and comparing this with the commonly used Stokes empirical equation [31]. As shown in Figure 11, the dropping velocity simulated using our algorithm increases first with gravity acceleration and then decreases as constrained by the bottom of the separator. The classical empirical Stokes equation assumes a constant force balance so that the velocity is a constant, but morphing and other constraints exist that impact the dropping velocity. Thus, the proposed numerical scheme is considered to be correct as it covers the simplified scenario, and it can provide more accurate information for separation engineering. Moreover, the mesh independence is also proved in the image, as the simulated dropping velocity using a coarse mesh (150×200) agree well the results of when a fine mesh is used.

This paper extends the study of oil–water membrane separations, incorporating the two-phase flow simulations in practical separators. Using our algorithm, a more accurate description of the penetration timing and velocity can be provided for further optimized designs of the membrane and optimization of practical operation plans. Moreover, the investigated temperature effect can suggest possible resolutions to various difficulties when separating oil and water in engineering contexts, for example, improvements to the separation performance of heavy oil productions. This idea has been validated in this paper by clear comparisons and was also accepted by our industrial partner with positive feedback from their engineering operations based on the simulation results. It should also be mentioned that in practical oil–water separators, there remain other factors that may challenge the certainty of the simulation, including heat transfer with the outer environment and other impurities such as asphalt. The effect of salinity may also cause scaling in the separator’s inner surface, which may further impact the fluid flow during the separation process.

## Figures and Tables

**Table 1:** Separation scenarios for each case.

Case Index	Oil Density	Water Density	Temperature ^1^
Case 1	0.8	1	T1
Case 2	0.8	1	T2
Case 3	0.8	1	T3
Case 4	0.75	1	T1
Case 5	0.9	1	T1

^1^  T2>T1>T3

**Table 2:** Separation scenarios for each case.

Case Index	Oil Density	Water Density	Temperature
Case 6	0.9	1	T2

## Data Availability

Not applicable.

## References

[1] Li Z.C., Fan C.L. (2020). A novel method to identify the flow pattern of oil–water two-phase flow. J. Pet. Explor. Prod. Technol..

[2] Zhang D., Zhang L., Tang H., Yuan S., Wang H., Chen S., Zhao Y. (2021). A novel fluid–solid coupling model for the oil–water flow in the natural fractured reservoirs. Phys. Fluids.

[3] Kong D., Gao Y., Sarma H., Li Y., Guo H., Zhu W. (2021). Experimental investigation of immiscible water-alternating-gas injection in ultra-high water-cut stage reservoir. Adv. Geo-Energy Res..

[4] Bai H. (2020). Mechanism analysis, anti-corrosion techniques and numerical modeling of corrosion in energy industry. Oil Gas Sci. Technol.–Rev. d’IFP Energ. Nouv..

[5] Hu P., Meng Q., Hu W., Shen F., Zhan Z., Sun L. (2016). A continuum damage mechanics approach coupled with an improved pit evolution model for the corrosion fatigue of aluminum alloy. Corros. Sci..

[6] Xia T., Feng Q., Wang S., Shu Q., Zhang Y., Sun Y. (2021). A numerical study of particle migration in porous media during produced water reinjection. J. Energy Resour. Technol..

[7] Liu L., Ciftci O.N. (2021). Effects of high oil compositions and printing parameters on food paste properties and printability in a 3D printing food processing model. J. Food Eng..

[8] Qin G., Zhang P., Wang Y. (2020). Investigating an assessment model of system oil leakage considering failure dependence. Environ. Sci. Pollut. Res..

[9] Appolinario L.R., Tschoeke D., Calegario G., Barbosa L.H., Moreira M.A., Albuquerque A.L.S., Thompson C.C., Thompson F.L. (2020). Oil leakage induces changes in microbiomes of deep-sea sediments of Campos Basin (Brazil). Sci. Total Environ..

[10] Wei Y., Qi H., Gong X., Zhao S. (2018). Specially wettable membranes for oil–water separation. Adv. Mater. Interfaces.

[11] Feng L., Zhang Z., Mai Z., Ma Y., Liu B., Jiang L., Zhu D. (2004). A super-hydrophobic and super-oleophilic coating mesh film for the separation of oil and water. Angew. Chem..

[12] Li X.M., Reinhoudt D., Crego-Calama M. (2007). What do we need for a superhydrophobic surface? A review on the recent progress in the preparation of superhydrophobic surfaces. Chem. Soc. Rev..

[13] Wang B., Liang W., Guo Z., Liu W. (2015). Biomimetic super-lyophobic and super-lyophilic materials applied for oil/water separation: A new strategy beyond nature. Chem. Soc. Rev..

[14] Li L., Xu Z., Sun W., Chen J., Dai C., Yan B., Zeng H. (2020). Bio-inspired membrane with adaptable wettability for smart oil/water separation. J. Membr. Sci..

[15] Hu J., Zhan Y., Zhang G., Feng Q., Yang W., Chiao Y.H., Zhang S., Sun A. (2021). Durable and super-hydrophilic/underwater super-oleophobic two-dimensional MXene composite lamellar membrane with photocatalytic self-cleaning property for efficient oil/water separation in harsh environments. J. Membr. Sci..

[16] Wang L., Zhang J., Wang S., Yu J., Hu W., Jiao F. (2020). Preparation of a polystyrene-based super-hydrophilic mesh and evaluation of its oil/water separation performance. J. Membr. Sci..

[17] Fernández E., Benito J.M., Pazos C., Coca J. (2005). Ceramic membrane ultrafiltration of anionic and nonionic surfactant solutions. J. Membr. Sci..

[18] Chiu T.Y., James A.E. (2006). Microfiltration of amphoteric surfactant using ceramic membranes. Colloids Surf. A Physicochem. Eng. Asp..

[19] Matos M., Gutiérrez G., Lobo A., Coca J., Pazos C., Benito J.M. (2016). Surfactant effect on the ultrafiltration of oil-in-water emulsions using ceramic membranes. J. Membr. Sci..

[20] Yang J., Zhang Z., Xu X., Zhu X., Men X., Zhou X. (2012). Superhydrophilic–superoleophobic coatings. J. Mater. Chem..

[21] Kobayashi M., Terayama Y., Yamaguchi H., Terada M., Murakami D., Ishihara K., Takahara A. (2012). Wettability and antifouling behavior on the surfaces of superhydrophilic polymer brushes. Langmuir.

[22] Tuteja A., Choi W., Ma M., Mabry J.M., Mazzella S.A., Rutledge G.C., Mckinley G.H., Cohen R.E. (2007). Designing superoleophobic surfaces. Science.

[23] Frank M., Kamenicky R., Drikakis D., Thomas L., Ledin H., Wood T. (2019). Multiphase flow effects in a horizontal oil and gas separator. Energies.

[24] Stewart A.C., Chamberlain N.P., Irshad M. (1998). A new approach to gas-liquid separation. European Petroleum Conference.

[25] Barton P.T., Drikakis D. (2010). An Eulerian method for multi-component problems in non-linear elasticity with sliding interfaces. J. Comput. Phys..

[26] Zhang T., Li Y., Li C., Sun S. (2020). Effect of salinity on oil production: Review on low salinity waterflooding mechanisms and exploratory study on pipeline scaling. Oil Gas Sci. Technol.–Rev. D’ifp Energ. Nouv..

[27] Zhang T., Sun S., Yu B. (2017). A fast algorithm to simulate droplet motions in oil/water two phase flow. Procedia Comput. Sci..

[28] Sun S., Zhang T. (2020). Reservoir Simulations: Machine Learning and Modeling.

[29] Shereshefsky J.L. (2002). Surface tension of saturated vapors and the equation of Eötvös. J. Phys. Chem..

[30] Dolgonosov A.M. (2016). The surface tension coefficients and critical temperatures of uniform nonpolar liquids from a priori calculations within the framework of the theory of generalized charges. Russ. Chem. Bull..

[31] Shearer S.A., Hudson J.R. (2008). Fluid Mechanics: Stokes’ Law and Viscosity. https://iicseonline.org/mechanics_and_fluids_1.pdf.

